# CLoDSA: a tool for augmentation in classification, localization, detection, semantic segmentation and instance segmentation tasks

**DOI:** 10.1186/s12859-019-2931-1

**Published:** 2019-06-13

**Authors:** Ángela Casado-García, César Domínguez, Manuel García-Domínguez, Jónathan Heras, Adrián Inés, Eloy Mata, Vico Pascual

**Affiliations:** Department of Mathematics and Computer Science, University of La Rioja, Ed. CCT. C/ Madre de Dios 53, Logroño, 26006 Spain

**Keywords:** Data augmentation, Classification, Detection, Segmentation, Multi-dimensional images

## Abstract

**Background:**

Deep learning techniques have been successfully applied to bioimaging problems; however, these methods are highly data demanding. An approach to deal with the lack of data and avoid overfitting is the application of *data augmentation*, a technique that generates new training samples from the original dataset by applying different kinds of transformations. Several tools exist to apply data augmentation in the context of image classification, but it does not exist a similar tool for the problems of localization, detection, semantic segmentation or instance segmentation that works not only with 2 dimensional images but also with multi-dimensional images (such as stacks or videos).

**Results:**

In this paper, we present a generic strategy that can be applied to automatically augment a dataset of images, or multi-dimensional images, devoted to classification, localization, detection, semantic segmentation or instance segmentation. The augmentation method presented in this paper has been implemented in the open-source package CLoDSA. To prove the benefits of using CLoDSA, we have employed this library to improve the accuracy of models for Malaria parasite classification, stomata detection, and automatic segmentation of neural structures.

**Conclusions:**

CLoDSA is the first, at least up to the best of our knowledge, image augmentation library for object classification, localization, detection, semantic segmentation, and instance segmentation that works not only with 2 dimensional images but also with multi-dimensional images.

## Background

Deep learning techniques are currently the state of the art approach to deal with bioimaging problems [1, 2]. However, these methods usually require a lot of data to work properly, and this might be a challenge in the bioimaging context. First of all, acquiring new data in problems related to, for instance, object recognition in biomedical images might be difficult [3–5]. Moreover, once the images have been acquired, they must be manually annotated, a task that is time-consuming and requires experts in the field to conduct it correctly [6].

A successful method that has been applied to deal with the problem of limited amount of data is *data augmentation* [7, 8]. This technique consists in generating new training samples from the original dataset by applying transformations that do not alter the class of the data. This method has been successfully applied in several contexts such as brain electron microscopy image segmentation [9], melanoma detection [3], or the detection of gastrointestinal diseases from endoscopical images [5]. Due to this fact, several libraries, like Augmentor [10] or Imgaug [11], and deep learning frameworks, like Keras [12] or Tensorflow [13], provide features for data augmentation in the context of object classification.

In general, those augmentation libraries have not been designed to deal with four common tasks in bioimaging problems: object localization (the identification of the position of an object in an image), object detection (the identification of the position of multiple objects in an image), semantic segmentation (predicting the class of each pixel of the image), and instance segmentation (generating a pixel-wise mask for every object in the image). These four problems can also take advantage from data augmentation [9, 14]; but, at least up to the best of our knowledge, it does not exist a general purpose library that can be applied to those problems and works with the standard annotation formats. This is probably due to the fact that, in the classification context, transformation techniques for image augmentation do not generally change the class of an image, but they might alter the annotation in the other four problems. For instance, applying the vertical flip operation to a melanoma image does not change the class of the image; but the position of the melanoma in the new image has changed from the original image. This means that, for each specific problem, special purpose methods must be implemented, or artificially generated images must be manually annotated. Neither of these two solutions is feasible when dealing with hundreds or thousands of images. In addition, augmentation libraries focus on datasets of 2 dimensional (2D) images, but do not deal with multi-dimensional images (such as z-stacks or videos).

In this paper, we present a generic method, see “Methods” section, that can be applied to automatically augment a dataset of images devoted to classification, localization, detection, semantic segmentation, and instance segmentation using the classical image augmentation transformations applied in object recognition; moreover, this method can be also applied to multi-dimensional images. Such a method has been implemented in an open-source library called CLoDSA that is introduced in “Implementation” section — the library, together with several examples and the documentation, is available at https://github.com/joheras/CLoDSA. We show the benefits of using CLoDSA when training models for different kinds of problems in “Results” section, and compare this library with other augmentation libraries in “Discussion” section. The paper ends with a section of conclusions and further work.

## Methods

**Fig. 1:**
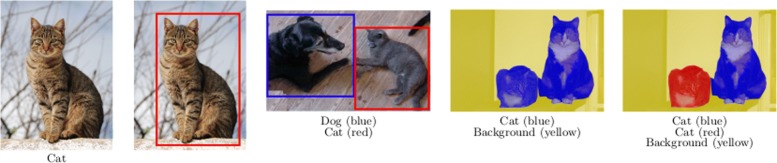
Examples of annotations, from left to right, for classification, localization, detection, semantic segmentation, and instance segmentation. Images obtained from the Oxford-IIIT Pet Dataset [17] which are available under a Creative Commons Attribution-ShareAlike 4.0 International License

In this section, we present an approach to augment images for the problems of object classification, localization, detection, semantic segmentation and instance segmentation. First of all, it is important to understand how the images are annotated in each of these five problems. In the case of object classification, each image is labeled with a prefixed category; for object localization, the position of the object in the image is provided using the bounding box (that is, the minimum rectangle containing the object); for object detection, a list of bounding boxes and the category of the objects inside those boxes are given; in semantic segmentation, each pixel of the image is labeled with the class of its enclosing object; and, finally in instance segmentation, each pixel of the image is labeled with the class of its enclosing object and objects of the same class are distinguished among them. An example of each kind of annotation is provided in Fig. 1. It is worth noting that, instance segmentation is the most general case, and the other problems can be seen as particular cases of such a problem; however, special purpose techniques and annotation formats have been developed to tackle each problem; and, therefore, we consider them separately.

Image augmentation for object classification is the simplest case. This task consists in specifying a set of transformations for which an image classification problem is believed to be invariant; that is, transformations that do not change the class of the image. It is important to notice that image-augmentation techniques are problem-dependent and some transformations should not be applied; for example, applying a 180 ^∘^ rotation to an image of the digit “6” changes its class to the digit “9”.

In the literature, the most commonly chosen image augmentation techniques for object classification are geometric transformations (such as translations, rotations, or scaling), color transformations (for instance, changing the color palette of the image or normalizing the image), filters (for example, Gaussian or median filters), and elastic distortions [8]. Other more specific techniques such as Generative Adversarial Networks (GANs) [15] have been also applied for image augmentation in object classification [16]; however, we will not consider GANs in our work since they cannot be directly applied for image augmentation in the other four problems.

For image augmentation in localization, detection, segmentation, and instance segmentation, we consider the classical image augmentation techniques applied in object classification, and split them into two categories. The former category consists of the techniques that leave invariant the position of the objects in the image; for example, changing the color palette of the image does not modify the position of an object. On the contrary, techniques that modify the position of the image belong to the latter category; for instance, rotation and translation belong to this category. A list of all the transformations that have been considered in this work, and their corresponding category, is available in Table 1.

**Table 1:** List of considered augmentation techniques

Position invariant techniques	Position variant techniques
Average blur	Crop
Bilateral blur	Elastic deformation
Brightness noising	Flip
Color noising	Rescale
Contrast noising	Rotation
Dropout	Skewing
Gamma correction	Translation
Gaussian blur	
Gaussian noise	
Hue jitter	
Median blur	
Normalization	
Random erasing	
Salt and pepper	
Saturation jitter	
Sharpen	
Value jitter	
Channel shift	
Lightning	
Change space color	

Image augmentation for localization, detection, segmentation, and instance segmentation using the techniques from the “invariant” category consists in applying the technique to the image and returning the resulting image and the original annotation as result. The rest of this section is devoted to explain, for each problem, how the annotation can be automatically generated for the techniques of the “variant” category.

**Fig. 2:**
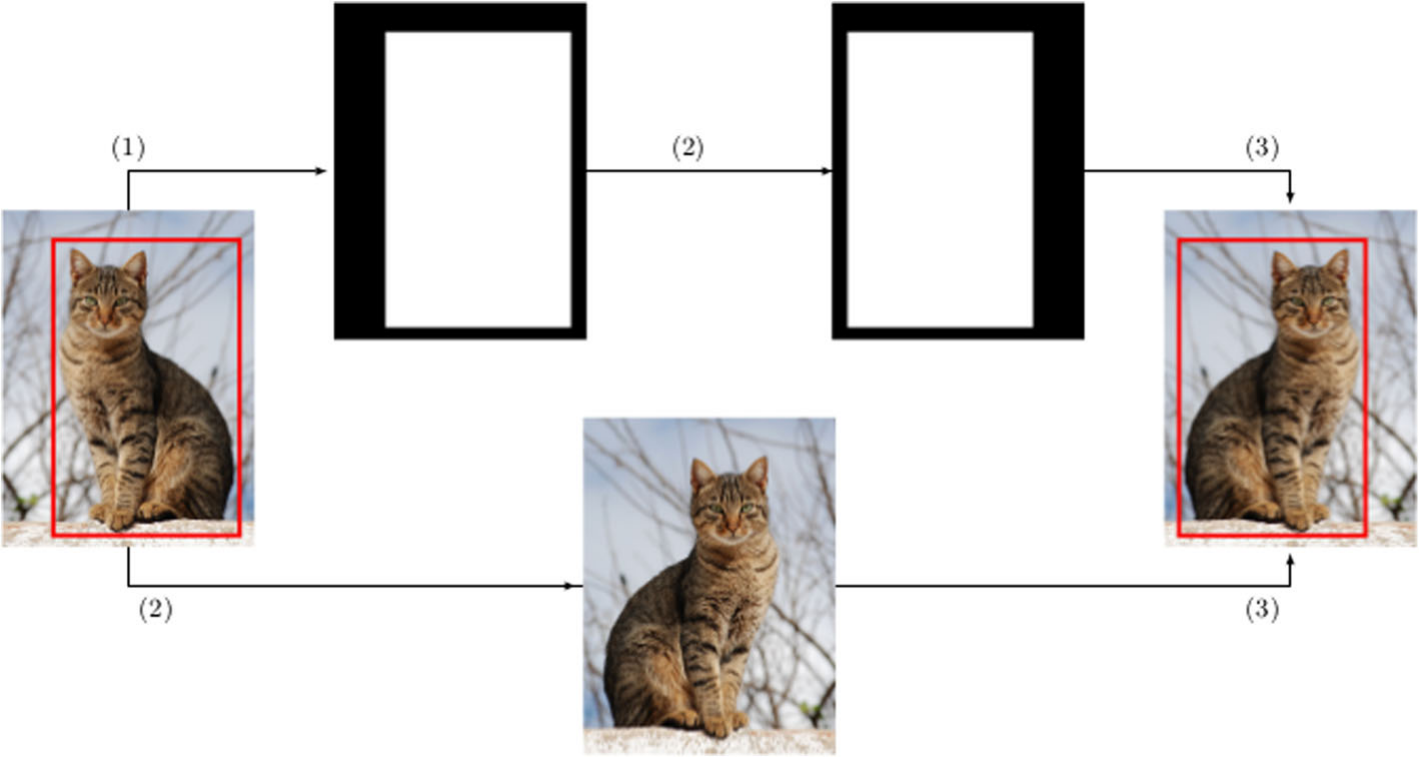
Process to automatically label augmented images for the localization problem: (1) generation of the mask, (2) application of the transformation operation (horizontal flip) to both the mask and the original image, and (3) combination of the bounding box containing the new mask and the transformed image

In the case of object localization, the first step to automatically generate the label from an annotated image consists in generating a mask from the annotated bounding box — i.e. a black image with a white rectangle indicating the position of the object. Subsequently, the transformation technique is applied to both the original image and the generated mask. Afterwards, from the transformed mask, the white region is simply located using basic contours properties, and the bounding box of the region is obtained — some transformations might generate a really small bounding box, or produce an image without bounding box at all since it will be located outside the boundaries of the image; to avoid that problem, a minimum percentage is required to keep the image; otherwise, the image is discarded. Finally, the transformed image is combined with the resulting bounding box to obtain the new annotated image. This process is depicted in Fig. 2 using as example the horizontal flip operation.

**Fig. 3:**
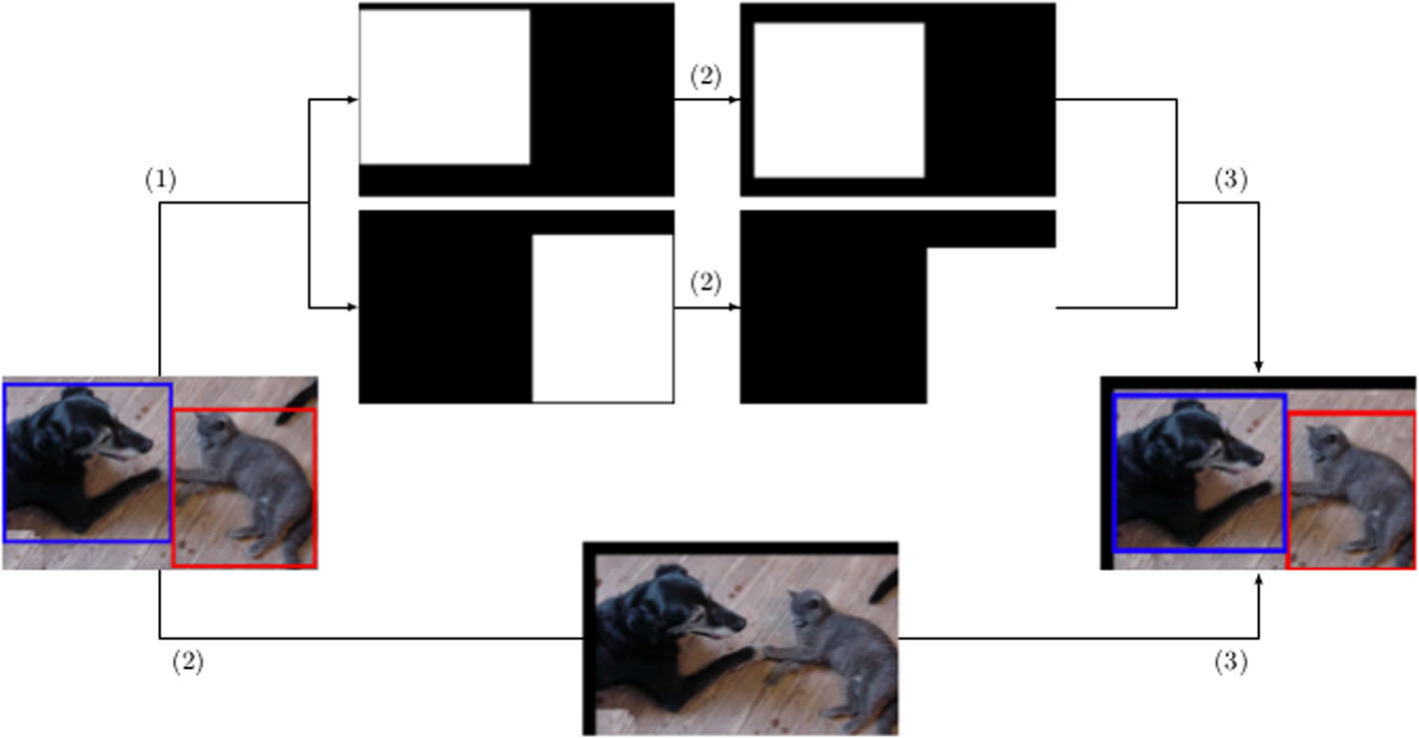
Process to automatically label augmented images for the detection problem: (1) generation of the masks, (2) application of the transformation operation (translation) to the masks and the original image, and (3) combination of the new masks and the transformed image

The procedure for image augmentation in object detection relies on the method explained for object localization. Namely, the only difference is that instead of generating a unique mask, a list of masks is generated for each bounding box of the list of annotations. The rest of the procedure is the same, see Fig. 3 using as example the translation operation.

**Fig. 4:**
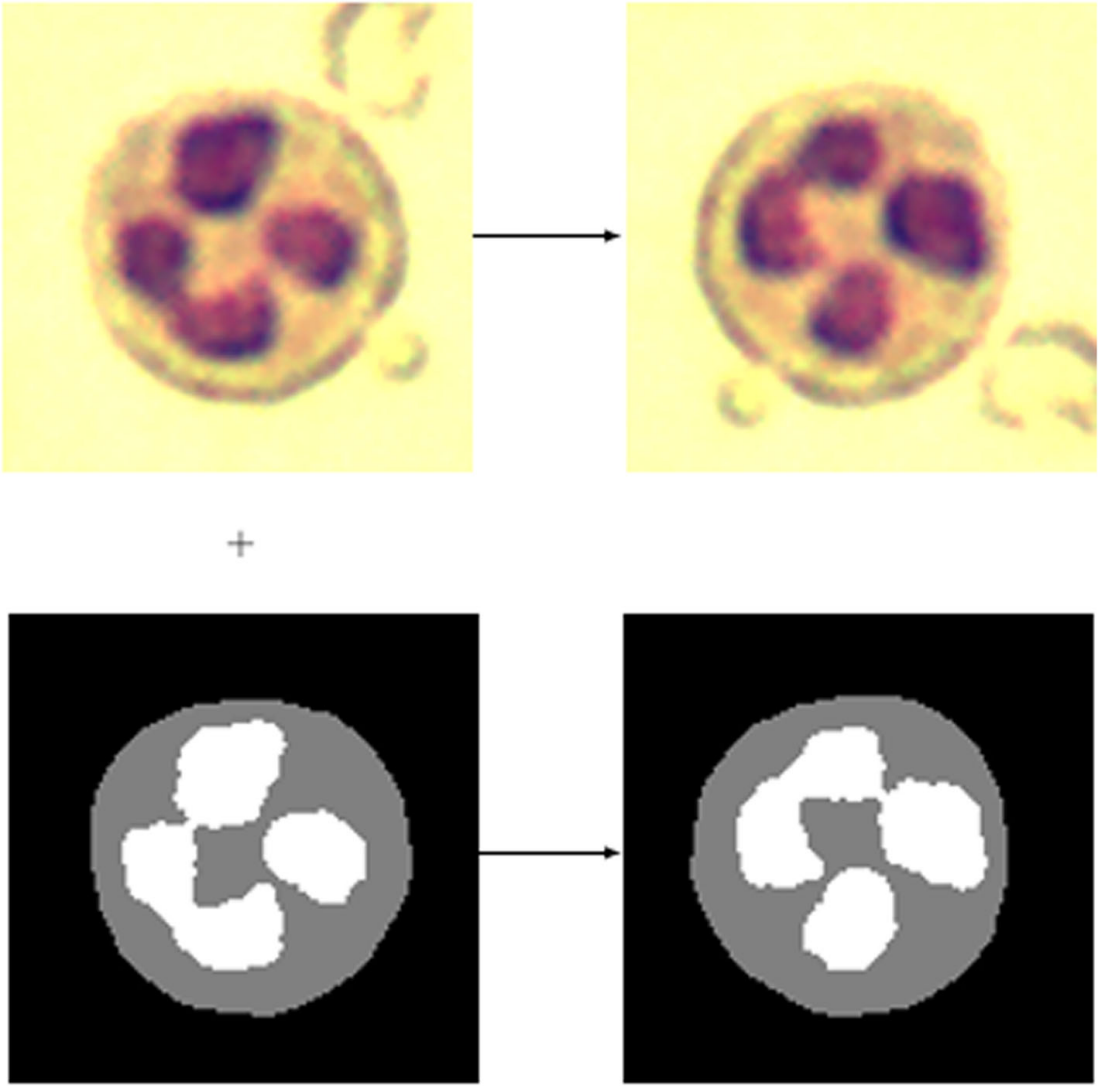
Process to automatically label augmented images for the semantic segmentation problem. From the original image (top left) and the annotation image (bottom left), two new images are generated by applying the transformation (in this case a 90^∘^ rotation) to both of them (top right and bottom right images). Images obtained from [20], these images are available under a Attribution-NonCommercial 3.0 Unported licence

In the semantic segmentation problem, given an image *I*, each pixel *I*_(*i*,*j*)_ of the image — i.e. the pixel of row *i* and column *j* of *I* — is labeled with the class of its enclosing object, this annotation is usually provided by means of an image *A* of the same size as the original image, where *A*_(*i*,*j*)_ provides the category of the pixel *I*_(*i*,*j*)_, and where each pixel category is given by a different value. In this case, the idea to automatically generate a new annotated image consists in applying the same transformation to the original and the annotation image, the result will be the combination of the two transformed images, see Fig. 4 where this procedure is shown using the rotation operation.

**Fig. 5:**
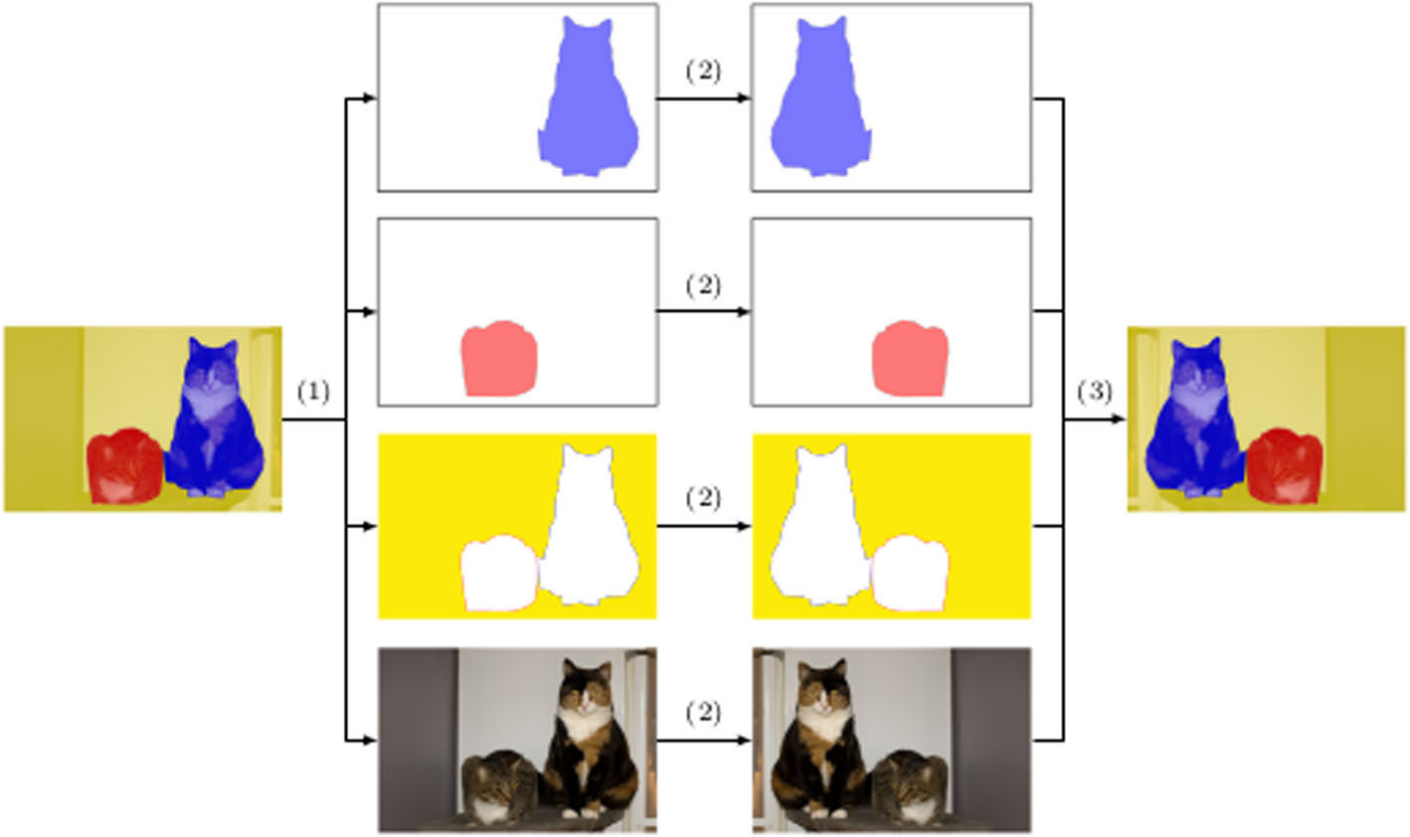
Process to automatically label augmented images for the instance segmentation problem. From the original annotated image (left), (1) the original image and a mask for each instance is obtained; (2) a vertical flip is applied to each image; and (3) the images are combined

Finally, we present the procedure for the instance segmentation problem. The idea is similar to the method explained for object detection. A mask is generated for each instance of the image. Subsequently, the transformation technique is applied to both the original image and the generated masks. Afterwards, from the transformed masks, the new instances are obtained. This process is depicted in Fig. 5.

The aforementioned procedures are focused on 2D images, but they can also be applied to multi-dimensional images that can be decomposed as a collection of images — this includes z-stacks and videos among others. The method consists in decomposing the multi-dimensional image into a collection of 2D images, applying the corresponding procedure, and finally combining back the resulting images into a multi-dimensional image.

## Implementation

The techniques presented in the previous section have been implemented as an open-source library called CLoDSA (that stands for Classification, Localization, Detection, Segmentation Augmentor). CLoDSA is implemented in Python and relies on OpenCV [18] and SciPy [19] to deal with the different augmentation techniques. The CLoDSA library can be used in any operating system, and it is also independent from any particular machine learning framework.

### CLoDSA configuration

CLoDSA augmentation procedure is flexible to adapt to different needs and it is based on six parameters: the dataset of images, the kind of problem, the input annotation mode, the output annotation mode, the generation mode, and the techniques to be applied. The dataset of images is given by the path where the images are located; and the kind of problem is either classification, localization, detection, segmentation, instance segmentation, stack classification, stack detection, or stack segmentation (the former five can be applied to datasets of 2D images, and the latter 3 to datasets of multi-dimensional images). The other four parameters and how they are managed in CLoDSA deserve a more detailed explanation.

The input annotation mode refers to the way of providing the labels for the images. CLoDSA supports the most-widely employed formats for annotating classification, localization, detection, semantic and instance segmentation tasks. For example, for object classification problems, the images can be organized by folders, and the label of an image be given by the name of the containing folder; another option for object classification labels is a spreadsheet with two columns that provide, respectively, the path of the image and the label; for object localization and detection there are several formats to annotate images such as the PASCAL VOC format [21] or the OpenCV format [22]; for semantic segmentation, the annotation images can be given in a devoted folder or in the same folder as the images; and, for instance segmentation, the COCO format is usually employed [23]. CLoDSA has been designed to manage different alternatives for the different problems, and can be easily extended to include new input modes that might appear in the future. To this aim, several design patterns, like the Factory pattern [24], and software engineering principles, such as dependency inversion or open/closed [25], have been applied. The list of input formats supported by CLoDSA for each kind of problem is given in Table 2 — a detailed explanation of the process to include new formats is provided in the project webpage.

**Table 2:** List of supported annotation formats

Data	Problem	Input format	Output format
2D Images	Classification	A folder for each class of image	A folder for each class of image
			An HDF5 file [26]
			A Keras generator [12]
	Localization	Pascal VOC format [21]	Pascal VOC format
			An HDF5 file
	Detection	Pascal VOC format	Pascal VOC format
		YOLO format [27]	YOLO format
	Segmentation	A folder containing the images and their associated masks	A folder containing the images and their associated masks
			An HDF5 file
			A Keras generator
	Instance segmentation	COCO format [23]	COCO format
		JSON format from ImageJ	JSON format from ImageJ
Multi-dimensional Images	Video Classification	A folder for each class of video	A folder for each class of video
	Video Detection	Youtube BB format [28]	Youtube BB format
	Stack segmentation	Pairs of tiff files containing the stack and the associated mask	Pairs of tiff files containing the stack and the associated mask

The output annotation mode indicates the way of storing the augmented images and their annotations. The first option can be as simple as using the same format or approach used to input the annotations. However, this might have the drawback of storing a large amount of images in the hard drive. To deal with this problem, it can be useful to store the augmented dataset using the standard Hierarchical Data Format (HDF5) [26] — a format designed to store and organize large amounts of data. Another approach to tackle the storage problem, and since the final aim of data augmentation is the use of the augmented images to train a model, consists in directly feeding the augmented images as batches to the model, as done for instance in Keras [12]. CLoDSA features these three approaches, and has been designed to easily include new methods in the future. The complete list of output formats supported by CLoDSA is given in Table 2.

The generation mode indicates how the augmentation techniques will be applied. Currently, there are only two possible modes: linear and power — in the future, new modes can be included. In the linear mode, given a dataset of *n* images, and a list of *m* augmentation techniques, each technique is applied to the *n* images producing at most *n*×*m* images. The power mode is a pipeline approach where augmentation techniques are chained together. In this approach, the images produced in one step of the pipeline are added to the dataset that will be fed in the next step of the pipeline producing a total of (2^*m*^−1)×*n* new images (where *n* is the size of the original dataset and *m* is the cardinal of the set of techniques of the pipeline).

Finally, the last but not least important parameter is the set of augmentation techniques to apply — the list of techniques available in CLoDSA is given in Table 1, and a more detailed explanation of the techniques and the parameters to configure them is provided in the project webpage. Depending on the particular problem, the CLoDSA users can select the techniques that are more fitted for their needs.

### The CLoDSA architecture

In order to implement the methods presented in “Methods” section, we have followed a common pattern applicable to all the cases: the Dependency Inversion pattern [24]. We can distinguished three kind of classes in our architecture: *technique* classes, that implement the augmentation techniques; *transformer* classes, that implement the different strategies presented in “Methods” section; and *augmentor* classes, that implement the functionality to read and save images and annotations in different formats. We explain the design of these classes as follows.

**Fig. 6:**
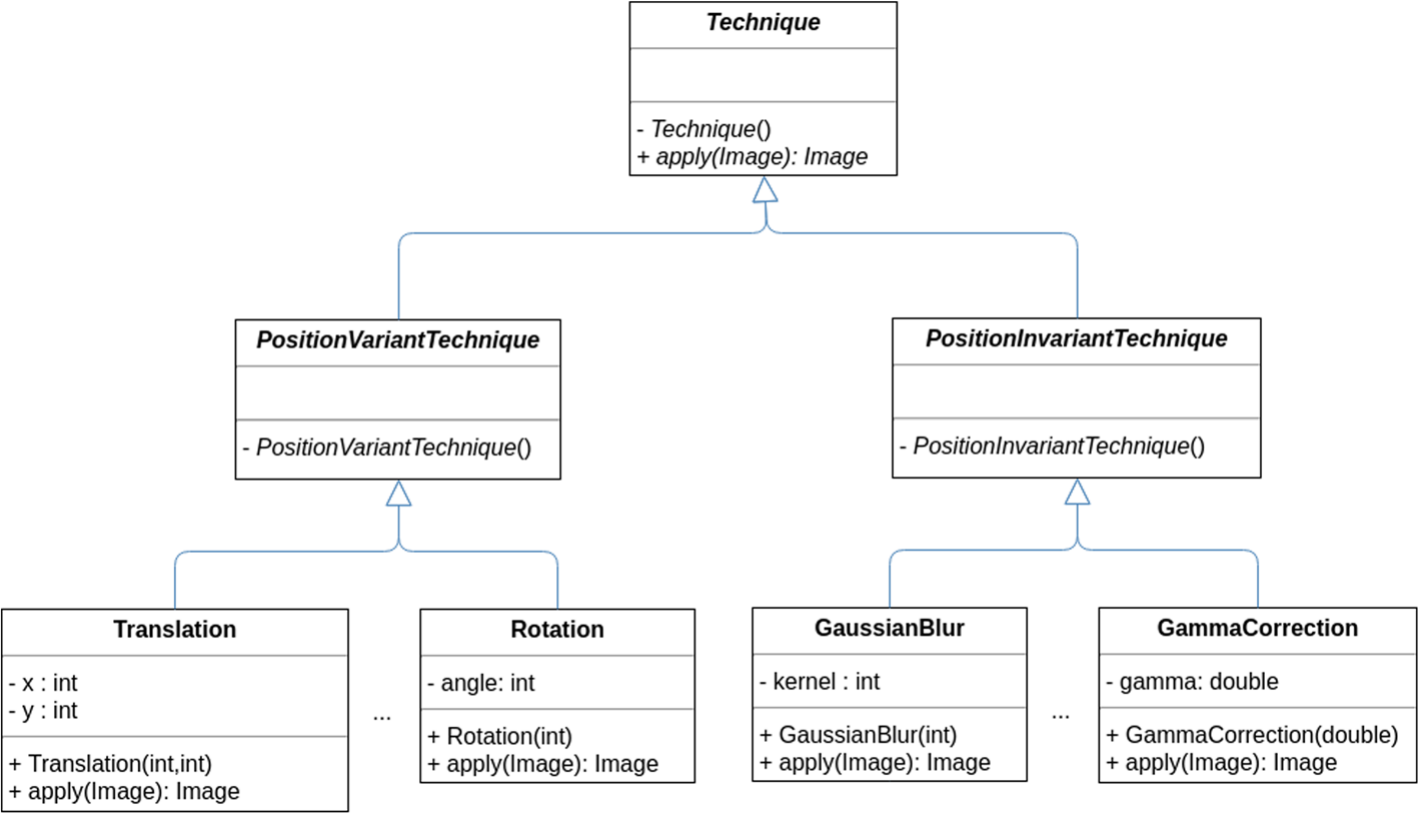
Simplification of the CLoDSA UML diagram for augmentation techniques

We have first defined an abstract class called *Technique* with two abstract subclasses called *PositionVariantTechnique* and *PositionInvariantTechnique* — to indicate whether the technique belongs to the position variant or invariant class — and with an abstract method called *apply*, that given an image produces a new image after applying the transformation technique. Subsequently, we have implemented the list of techniques presented in Table 1 as classes that extend either the *PositionVariantTechnique* or the *PositionInvariantTechnique* class, see Fig. 6.

**Fig. 7:**
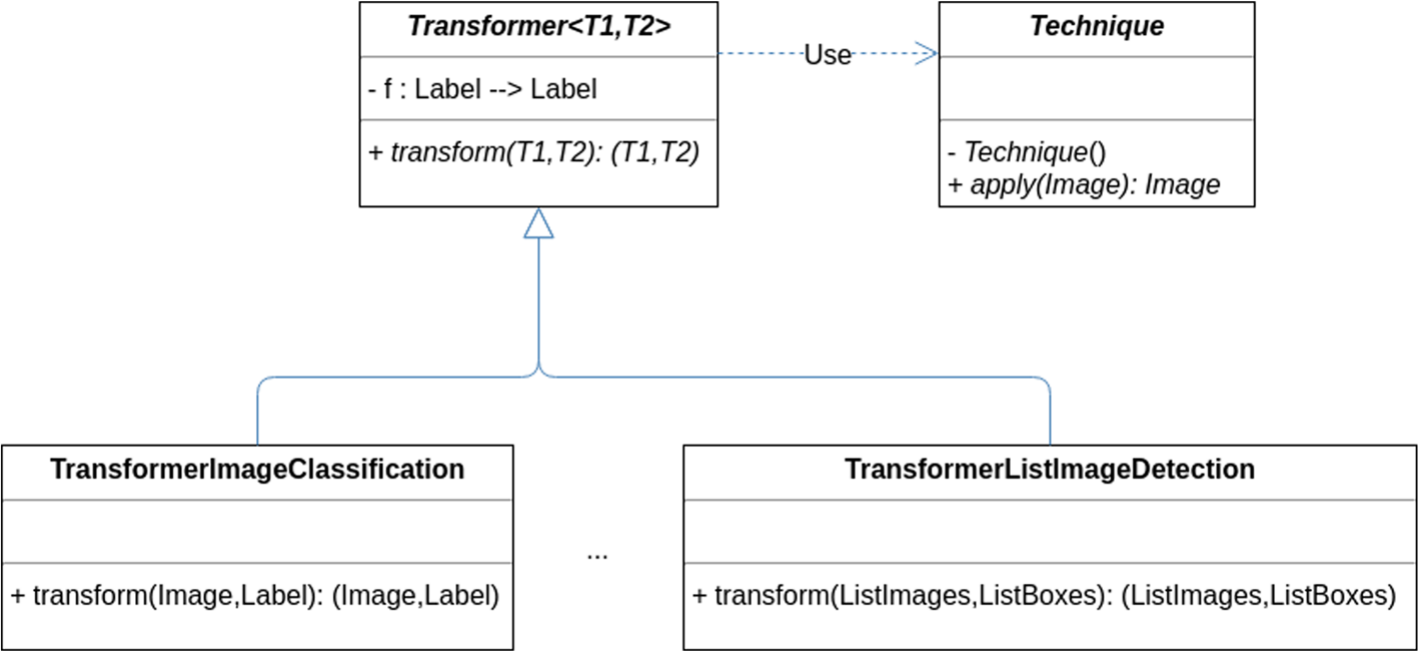
Simplification of the CLoDSA UML diagram for transformers

Subsequently, we have defined a generic abstract class [29] called *Transformer* <*T*_1_,*T*_2_>, where *T*_1_ represents the type of data (2D or multi-dimensional images) to be transformed, and *T*_2_ represents the type of the annotation for *T*_1_; for example, a box or a mask — the concrete types are fixed in the concrete classes extending the abstract class. This abstract class has two parameters, an object of type *Technique*, and a function *f* from *label* to *label*; and an abstract method called *transform* that given a pair (*T*_1_,*T*_2_) (for instance, in object detection, an image and a list of boxes indicating the position of the objects in the image) produces a new pair (*T*_1_,*T*_2_) using one of the augmentation strategies presented in “Methods” section — the strategy is implemented in the subclasses of the *Transformer* <*T*_1_,*T*_2_> class. The purpose of the function *f* is to allow the *transform* method to not only change the position of the annotations but also their associated class. As we have previously mentioned, in general, data augmentation procedures apply techniques that do not change the class of the objects of the image; but there are cases when the transformation technique changes the class (for instance, if we have a dataset of images annotated with two classes, people looking to the left and people looking to the right, applying a vertical flip changes the class); the function *f* encodes that modification — by default, this function is defined as the identity function. This part or the architecture is depicted in Fig. 7.

**Fig. 8:**
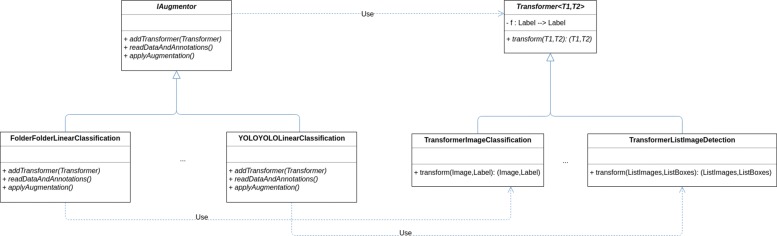
Simplification of the CLoDSA UML diagram for augmentors

Finally, we have defined an interface called *IAugmentor* that has three methods *addTransformer*, *readDataAndAnnotations*, and *applyAugmentation*; see Fig. 8. The classes implementing this interface are in charge of reading the data and annotations in a concrete format (using the *readDataAndAnnotations*), applying the augmentation (by means of the *applyAugmentation* and using objects of the class *Transformer* injected using the *addTransformer* method), and storing the result — the input and output format available are indicated in Table 2. In order to ensure that the different objects of the architecture are constructed properly (that is, satisfying the required dependencies) the Factory pattern has been employed [24].

Therefore, using this approach, the functionality of CLoDSA can be easily extended in several ways. It is possible to add new augmentation techniques by adding new classes that extend the *Technique* class. Moreover, we can also extend the kinds of problems that can be tackled in CLoDSA by adding new classes that extend the *Transformer* class. Finally, we can manage new input/output formats by providing classes that implement the *IAugmentor* interface. Several examples showing how to include new functionality in CLoDSA can be found in the project webpage.

### Using CLoDSA

We finish this section by explaining the different modes of using CLoDSA. This library can be employed by both expert and non-expert users.

First of all, users that are used to work with Python libraries can import CLoDSA as any other library and use it directly in their own projects. Several examples of how the library can be imported and employed are provided in the project webpage. This kind of users can extend CLoDSA with new augmentation techniques easily. The second, and probably the most common, kind of CLoDSA’s users are researchers that know how to employ Python but do not want to integrate CLoDSA with their own code. In this case, we have provided several Jupyter notebooks to illustrate how to employ CLoDSA for data augmentation in several contexts — again the notebooks are provided in the project webpage and also as supplementary materials. An example of this interaction is provided in Appendix A.

CLoDSA can be also employed without any knowledge of Python. To this aim, CLoDSA can be executed as a command line program that can be configured by means of a JavaScript Object Notation (JSON) file [30]. Therefore, users who know how to write JSON files can employ this approach. Finally, and due to the fact that the creation of a JSON file might be a challenge for some users since there is a great variety of options to configure the library; we have created a step-by-step Java wizard that guides the user in the process of creating the JSON file and invoking the CLoDSA library. In this way, the users, instead of writing a JSON file, select in a simple graphical user interface the different options for augmenting their dataset of images, and the wizard is in charge of generating the JSON file and executing the augmentation procedure. Besides, since new configuration options might appear in the future for CLoDSA, the Java wizard can include those options by modifying a configuration file — this avoids the issue of modifying the Java wizard every time that a new option is included in CLoDSA.

## Results

To show the benefits of applying data augmentation using CLoDSA, we consider three different bioimaging datasets as case studies.

### Malaria parasite classification

The first case study focuses on an image classification problem. To this aim, we consider the classification of Malaria images [31], where images are labelled as parasitized or uninfected; and, we analyse the impact of applying data augmentation when constructing models that employ transfer-learning [32].

**Fig. 9:**
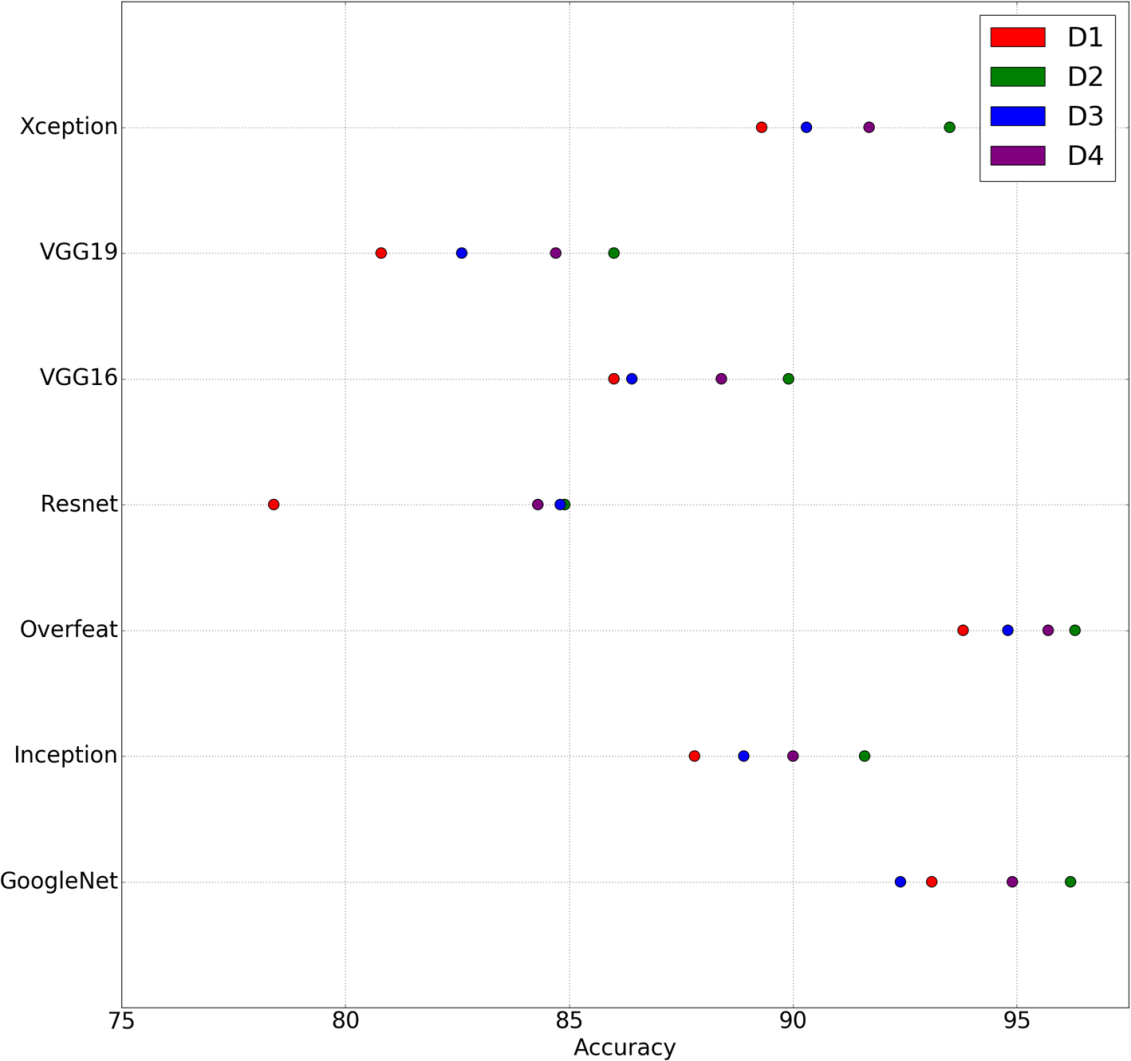
Scatter plot showing the accuracy of the models constructed for the different versions of the Malaria dataset (where *D*_1_ is the original dataset; and *D*_2_, *D*_3_ and *D*_4_ are the augmented datasets) using different feature extractor methods

Transfer learning is a deep learning technique that consists in partially re-using a deep learning model trained in a source task in a new target task. In our case, we consider 7 publicly available networks trained on the ImageNet challenge [33] (the networks are GoogleNet [34], Inception v3 [35], OverFeat [36], Resnet 50 [37], VGG16 [38], VGG19 [38], and Xception v1 [39]) and use them as feature extractors to construct classification models for the Malaria dataset. For each feature extractor network, we consider 4 datasets: *D*_1_ is the original dataset that consists of 1000 images (500 images per class); *D*_2_ was generated from *D*_1_ by applying flips and rotations (*D*_2_ consists of 5000 images, the original 1000 images and 4000 generated images); *D*_3_ was generated from *D*_1_ by applying gamma correction and equalisation of histograms (*D*_3_ consists of 3000 images, the original 1000 images and 2000 generated images); and, *D*_4_ is the combination of *D*_2_ and *D*_3_ (*D*_4_ consists of 7000 images, the original 1000 images and 6000 generated images). In order to evaluate the accuracy of the models, a stratified 5-fold cross-validation approach was employed using the FrImCla framework [40] (a tool for constructing image classification models using transfer learning), and the results are shown in Fig. 9.

As can be seen in the scatter plot of Fig. 9, the accuracy of the models constructed for each feature extractor method increases when data augmentation is applied. The improvement ranges from a 0.4*%* up to a 6.5*%*; and, there is only one case where applying data augmentation has a negative impact on the accuracy of the model. Moreover, we can notice that we obtain better models only applying flips and rotations (dataset *D*_2_) than using a bigger dataset where we have applied not only flips and rotations but also color transformations (dataset *D*_4_). This indicates the importance of correctly selecting the set of data augmentation techniques — an active research area [41–43].

### Stomata detection

In the second example, we illustrate how CLoDSA can be employed to augment a dataset of images devoted to object detection, and the improvements that are achieved thanks to such an augmentation. In particular, we have trained different models using the YOLO algorithm [27] to detect stomata in images of plant leaves — stomata are the pores on the plant leaf that allow the exchange of gases.

For this case study, we have employed a dataset of 131 stomata images that was split into a training set of 100 images (from now on *D*_1_), and a test set of 31 images. The dataset *D*_1_ was augmented using three approaches: applying different kinds of flips (this dataset is called *D*_2_ and contains 400 images); applying blurring, equalisation of histograms and gamma correction (this dataset is called *D*_3_ and contains 400 images); and, combining *D*_2_ and *D*_3_ (this dataset is called *D*_4_ and contains 700 images).

Using each one of the four datasets, a YOLO model was trained for 100 epochs; and, the performance of those models in the test set, and using different metrics, is shown in Table 3. As can be seen in that table, the models that are built using the augmented datasets produce much better results. In particular, the precision is similar in all the models, but the recall and F1-score of the augmented datasets are clearly higher (for instance, the F1-score goes from 75% in the original dataset to 97% in *D*_3_). As in the previous case study, one of the models constructed from smaller datasets (namely, *D*_3_) produces better results that the one built with a bigger dataset (*D*_4_). This again shows the importance of having a library that easily allows to generate different datasets of augmented images.

**Table 3:** Results using YOLO models trained with different datasets (*D*_1_ is the original dataset, *D*_2_ is *D*_1_ augmented using flips; *D*_3_ is *D*_1_ augmented using blurring, gamma correction and equalisation; and *D*_4_ is the combination of *D*_2_ and *D*_3_) for the stomata dataset

	Precision	Recall	F1-score	TP	FP	FN	IoU
*D*_1_	0.97	0.61	0.75	591	21	374	0.75
*D*_2_	0.97	0.88	0.92	852	26	113	0.81
*D*_3_	0.95	1.00	0.97	961	52	4	0.79
*D*_4_	0.99	0.90	0.94	869	12	96	0.83

### Semantic segmentation of neural structures

Finally, we show how CLoDSA can improve results in semantic segmentation tasks. In particular, we tackle the automatic segmentation of neural structures using the dataset from the ISBI challenge [44]. In this challenge, the dataset consists of 30 images (512×512 pixels) from serial section transmission electron microscopy of the Drosophila first instar larva ventral nerve cord. Each image is annotated with a ground truth segmentation mask where white pixels represents cells and black pixels membranes.

From the dataset of 30 images, we split the dataset into a training set containing 20 images (we call this dataset *D*_1_), and a test set with the remaining images. We augmented the dataset *D*_1_ using CLoDSA in three different ways. First of all, we constructed a dataset *D*_2_ from *D*_1_ by applying elastic deformations (the dataset *D*_2_ contains 40 images, the 20 original images of *D*_1_ and 20 generated images). In addition, we built a dataset *D*_3_ from *D*_1_ by applying geometric and colour transformations (namely, rotations, translations, flips, shears, gamma correction and equalizations) — the dataset *D*_3_ contains 220 images, the 20 original images of *D*_1_ and 200 generated images. And, finally, a dataset *D*_4_ was constructed by combining the datasets *D*_2_ and *D*_3_ (the dataset *D*_4_ contains 240 images since the images of *D*_1_ are only included once).

From these four datasets, we have trained four different models using the U-Net architecture [14] for 25 epochs. Those models have been evaluated using the test set and considering as metrics the accuracy, the F1-score, the precision, the recall, the specificity, and the balanced accuracy. The results are shown in Table 4. Since the number of white pixels and black pixels in the mask images are imbalanced, the most interesting metric is the balanced accuracy (that combines the recall and the specificity); and, as we can see from Table 4, by applying data augmentation we can improve the results of our models.

**Table 4:** Results using several models trained with different datasets (*D*_1_ is the original dataset, *D*_2_ is *D*_1_ augmented using elastic deformations, *D*_3_ is *D*_1_ augmented using geometric and color transformations, and *D*_4_ is the combination of *D*_2_ and *D*_3_) for the ISBI challenge

	Accuracy	F1-score	Precision	Recall	Specificity	Balanced accuracy
*D*_1_	0.90	0.94	0.93	0.94	0.76	0.85
*D*_2_	0.90	0.94	0.94	0.94	0.77	0.855
*D*_3_	0.90	0.94	0.95	0.92	0.82	0.87
*D*_4_	0.91	0.94	0.94	0.94	0.78	0.86

## Discussion

Image augmentation techniques have been successfully applied in the literature; and most of those techniques can be directly implemented using image processing and computer vision libraries, such as OpenCV or SciPy, or even without the help of third-party libraries. However, this means reinventing the wheel each time; and, hence, several libraries and frameworks have appeared over the years to deal with image augmentation for object classification.

Some of those libraries, like Data-Augmentation [45] or CodeBox [46], provide a few basic augmentation techniques such as rotation, shifting and flipping. There are other libraries with more advanced features. Augmentor [10] uses a stochastic, pipeline-based approach for image augmentation featuring the most common techniques applied in the literature. Imgaug [11] provides more than 40 augmentation techniques, and albumentations [47] is the fastest augmentation library. CLoDSA includes almost all the augmentation techniques implemented in those libraries and also others that have been employed in the literature but were not included in those libraries. A comparison of the techniques featured in each library is available in the project webpage, and also as a supplementary material.

All the aforementioned libraries are independent from any particular machine learning framework, but there are also image augmentation libraries integrated in several deep learning frameworks. The advantage of those libraries is that, in addition to save the images to disk, they can directly fed the augmented images to a training model without storing them. The main deep learning frameworks provide data augmentation techniques. Keras can generate batches of image data with real-time data augmentation using 10 different augmentation techniques [48]. There is a branch of Caffe [49] that features image augmentation using a configurable stochastic combination of 7 data augmentation techniques [50]. Tensorflow has TFLearn’s DataAugmentation [51], MXNet has Augmenter classes [52], DeepLearning4J has ImageTransform classes [53], and Pytorch has transforms [54].

In addition to these integrated libraries for image augmentation, the Augmentor library, that can be used independently of the machine learning framework, can be integrated into Keras and Pytorch. This is the same approach followed in CLoDSA where we have developed a library that is independent of any framework but that can be integrated into them — currently such an integration is only available for the Keras framework.

Most of those libraries, both those that are independent of any framework, and those that are integrated into a deep learning library, are focused on the problem of object classification, and only Imgaug and albumentations can be applied to the problems of localization, detection, semantic segmentation and instance segmentation. CLoDSA can be used for dataset augmentation in problems related to classification, localization, detection, semantic segmentation, and instance segmentation; and, additionally brings to the table several features that are not included in any other library.

The main difference between CLoDSA and the libraries Imgaug and albumentations in the problems related to localization, detection, semantic segmentation, and instance segmentation is the way of handling the annotations. The annotations of the images in Imgaug or albumentations must be coded inside Python before using them for the augmentation process; on the contrary, CLoDSA deals with the standard formats for those imaging problems. From the users point of view, the CLoDSA approach is simpler since they can directly use the annotation files generated from annotation tools (for instance, LabelImg [55], an annotation tool for object detection problems, or the Visipedia Annotation Toolkit for image segmentation [56]) and that can be latter fed to deep learning algorithms.

Another feature only available in CLoDSA is the chance of automatically changing the class of an object after applying a transformation technique. This feature can be applied not only when augmenting images for object classification, but also for the other problems supported by CLoDSA. Finally, image augmentation libraries are focused on 2D images; on the contrary, CLoDSA not only works with this kind of images, but can also apply augmentation techniques to multi-dimensional images that can be decomposed in a collection of images (such as stacks of images, or videos). As in the case of 2D images, CLoDSA can augment those multi-dimensional images for the classification, localization, detection, semantic segmentation, and instance segmentation problems.

## Conclusions and further work

In this work, we have presented an approach that allows researchers to automatically apply image augmentation techniques to the problems of object classification, localization, detection, semantic segmentation, and instance segmentation. Such a method works not only with 2D images, but also with multi-dimensional images (such as stacks or videos). In addition, the method has been implemented in CLoDSA. This library has been designed using several object oriented patterns and software engineering principles to facilitate its usability and extensibility; and the benefits of applying augmentation with this library have been proven with three different datasets.

In the future, we plan to expand the functionality of CLoDSA to include more features; for example, generate images using a stochastic pipeline approach as in [10], include more augmentation techniques, or integrate it into more deep learning frameworks. Another task that remains as further work is the definition of a method that could employ GANs to augment images for the problems of localization, detection, semantic segmentation and instance segmentation.

## Appendix A: A coding example

Let us consider that we want to augment a dataset of images for object detection using the annotation format employed by the YOLO detection algorithm [27] — in this format, for each image a text file containing the position of the objects of such an image is provided. The dataset is stored in a folder called *yoloimages*, and we want to apply three augmentation techniques: vertical flips, random rotations, and average blurring. After loading the necessary libraries, the user must specify the six parameters explained in “Implementation” section (the path to the dataset of images, the kind of problem, the input annotation mode, the output annotation mode, the generation mode, and the techniques to be applied). We store those values in the following variables.


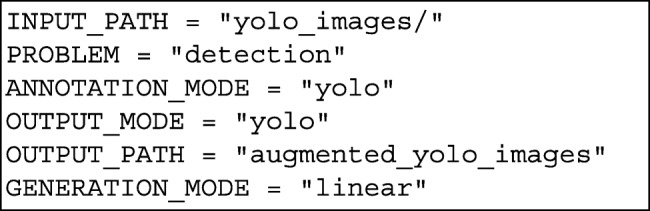


Subsequently, we define an augmentor object that receives as parameters the above variables.


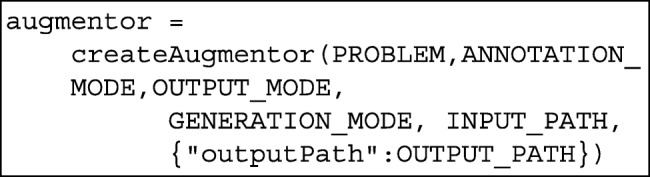


The above function uses the Factory pattern to construct the correct object, but the user does not need to know the concrete class of the object.

Afterwards, we define the augmentation techniques and add them to the augmentor object using a transformer object.


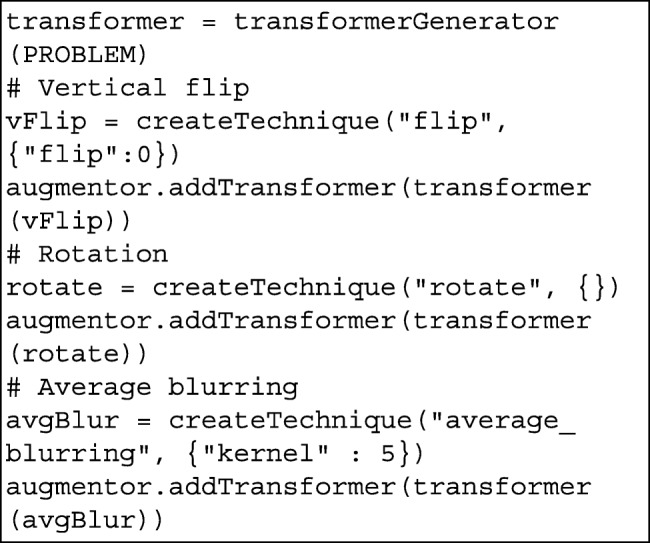


Finally, we invoke the applyAugmentation method of the augmentor object to initiate the augmentation process:


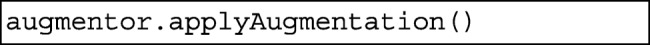


After a few seconds (depending on the initial amount of images), the new images and their corresponding annotations will be available in the output folder.

## Data Availability

The source code of CLoDSA is available at https://github.com/joheras/CLoDSA, where the interested reader can also find the data for the experiments presented in the “Results” section. CLoDSA can be installed as a Python library using pip, and it is distributed using the GNU GPL v3 license.

## Abbreviations

2D: 2 dimensional
GANs: Generative adversarial networks
HDF5: Hierarchical data format
JSON: JavaScript object notation
